# Cheetahs, *Acinonyx jubatus*, balance turn capacity with pace when chasing prey

**DOI:** 10.1098/rsbl.2013.0620

**Published:** 2013-10-23

**Authors:** John W. Wilson, Michael G. L. Mills, Rory P. Wilson, Gerrit Peters, Margaret E. J. Mills, John R. Speakman, Sarah M. Durant, Nigel C. Bennett, Nikki J. Marks, Michael Scantlebury

**Affiliations:** 1 Department of Biology, North Carolina State University, Raleigh, NC 27695, USA; 2 The Lewis Foundation, PO Box 411703, Craighall 2024, South Africa; 3 WildCRU, Zoology Department, University of Oxford, The Recanati-Kaplan Centre, Tubney House, Abingdon OX13 5QL, UK; 4 Swansea Laboratory for Animal Movement, Biosciences, College of Science, Swansea University, Singleton Park, Swansea SA2 8PP, UK; 5 earth&OCEAN Technologies, Krummbogen 32, 24113 Kiel, Germany; 6 Institute of Biological and Environmental Sciences, University of Aberdeen, Aberdeen, Scotland AB24 2TZ, UK; 7 Institute of Zoology, Zoological Society of London, Regents Park, London NW1 4RY, UK; 8 Mammal Research Institute, University of Pretoria, Pretoria 0002, South Africa; 9 School of Biological Sciences, Queen's University Belfast, Belfast BT9 7BL, Northern Ireland, UK

**Keywords:** acceleration, energy, movement, predator, speed, turning

## Abstract

Predator–prey interactions are fundamental in the evolution and structure of ecological communities. Our understanding, however, of the strategies used in pursuit and evasion remains limited. Here, we report on the hunting dynamics of the world's fastest land animal, the cheetah, *Acinonyx jubatus*. Using miniaturized data loggers, we recorded fine-scale movement, speed and acceleration of free-ranging cheetahs to measure how hunting dynamics relate to chasing different sized prey. Cheetahs attained hunting speeds of up to 18.94 m s^−1^ and accelerated up to 7.5 m s^−2^ with greatest angular velocities achieved during the terminal phase of the hunt. The interplay between forward and lateral acceleration during chases showed that the total forces involved in speed changes and turning were approximately constant over time but varied with prey type. Thus, rather than a simple maximum speed chase, cheetahs first accelerate to decrease the distance to their prey, before reducing speed 5–8 s from the end of the hunt, so as to facilitate rapid turns to match prey escape tactics, varying the precise strategy according to prey species. Predator and prey thus pit a fine balance of speed against manoeuvring capability in a race for survival.

## 1. Introduction

The interactions between predators and prey are fundamental for the evolution and structure of ecological communities [1]. Our understanding, however, of the strategies adopted by predators and prey during pursuit and evasion remains limited. Recent advances in the miniaturization of animal-borne sensors now enable us to measure the fine-scale movements of free-ranging animals with hitherto unparalleled accuracy. Here, we use miniaturized data loggers to document hunting strategies of the cheetah *Acinonyx jubatus*. We report that, besides the oft-cited power costs for forward acceleration [2], turning costs to follow ‘jinking’ prey may also play a critical role in hunting strategy, necessitating speed modulation. To our knowledge, this is the first time that fine-scale hunting strategies of any terrestrial predator have been documented (but cf. [3] for marine predators).

Cheetahs are morphologically [4,5] and physiologically [6,7] adapted to running, being capable of attaining speeds in excess of 28 m s^−1^ [2,8–10]. Such high speeds should enable them to run down slower prey, with failed hunts attributed to exhaustion [7] or overheating [11]. However, prey escape tactics can involve sudden directional changes [9,12,13], which are more difficult to accommodate with increasing velocity [2]. Moreover, turns at higher speeds lead to greater forces on animals' limbs and muscles, particularly when turn angles are acute [14,15], as well as higher energetic costs [16]. Thus, while the ability to hunt at high speed may enable cheetahs to outrun prey, they may not always choose to use maximum speed, especially when chasing prey that attempts evasion by sudden changes in direction.

To examine the interplay of speed and turning, we deployed GPS and accelerometer loggers on six free-ranging cheetahs in Kgalagadi Transfrontier Park, southern Africa, to measure how hunt trajectory, speed and acceleration related to different prey species chased.

## 2. Material and methods

Speed, position and acceleration estimates were obtained using MiniGPS devices (earth&OCEAN Technologies, Germany) and accelerometer loggers (Cefas G6A, UK) attached to drop-off collars (SIRTRACK, New Zealand). Two or three GPS units and two accelerometers were deployed per animal. GPS devices, each lasting 9–12 h, were programmed to obtain positional fixes at 1.0 Hz over consecutive days. Accelerometers recorded at 30 Hz in each of the three orthogonal axes over 5.5 days.

We calculated linear acceleration as the change in speed and angular velocity, as the change in geometrically calculated angle between sequential GPS fixes. Raw accelerometer data were converted to static body acceleration (SBA) by smoothing each channel using a running mean of 2 s; axis-specific dynamic body acceleration (DBA) was then derived by subtracting axis-specific static acceleration from the raw data [17].

Vectorial dynamic body acceleration (VeDBA) and vectorial static body acceleration (VeSBA) were calculated as the vectorial sum of the three DBA and SBA axes, respectively [18]. Under conditions of constant velocity, VeSBA = 1.0*g*, whereas a departure from 1.0*g* (*ΔS*) indicates the *g*-force derived from forward and sideways acceleration produced by the cheetahs during their chases, independent of gravity [19]. From Newton's law, where force = mass × acceleration, any deviation from 1.0*g* indicates that the (terrestrial) animal is exerting a force, which will vary with speed and turn radius [14,15], with a consequent energetic cost [16] (For a detailed description of accelerometer and GPS devices, and analyses, see electronic supplementary material, S1).

## 3. Results

We recorded movements using GPS devices for six chases during a total logger-active period of 124 h. From visual observations, prey species comprised one large ostrich chick (*Struthio camelus*) which was captured, three adult steenbok (*Raphicerus campestris*), two of which were captured, and two springbok (*Antidorcas marsupialis*) which were not captured. With accelerometers, we recorded an additional 35 chases over 1375 h; additional prey species included hare (*Lepus* spp.), common duiker (*Sylvicapra grimmia*), blue wildebeest (*Connochaetes taurinus*) calf and gemsbok (*Oryx gazella*) calf.

All chases were brief, with the longest run lasting 59 s and the greatest period of continuous running in excess of 13.9 m s^−1^ lasting 23 s and covering 379 m. The fastest speed was 18.94 m s^−1^ and the fastest (GPS-derived) linear acceleration was 7.5 m s^−2^, while the highest (accelerometer-derived) VeDBA was 4.70*g*.

**Figure 1.**
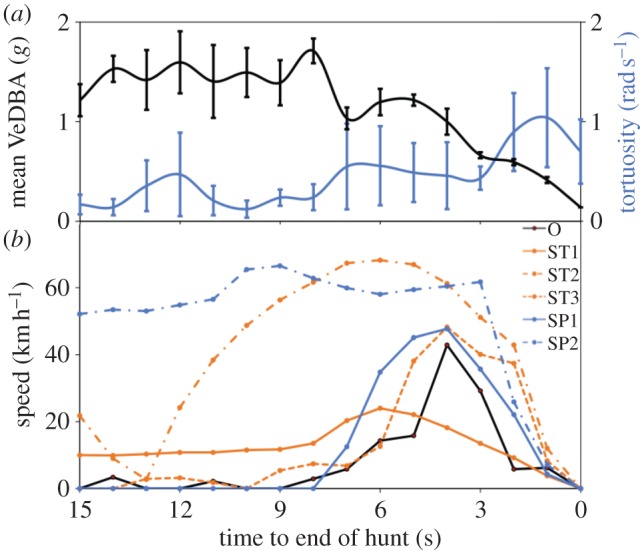
(*a*) Mean VeDBA (*g*, 9.81 m s^−2^) and angular velocity (rad s^−1^) against time (*n* = 35, 30 Hz) during the last 15 s of cheetah chases. Error bars represent standard errors; (*b*) Speed (km h^−1^) against time (s) (*n* = 6, 1.0 Hz) across the last 15 s of cheetah chases for ostrich (O), steenbok (ST1, ST2 and ST3) and springbok (SP1 and SP2). Hunts O, ST2 and ST3 were successful.

Overall, the faster cheetahs ran, the less tortuous route they took (*F*_1,176_ = 4.32, *p* = 0.039; figure 1) with angular velocity differing in response to prey (*χ*^2^ = 12.25, *p* = 0.032). The mean of the greatest speeds attained in individual hunts was 12.90 m s^−1^, and occurred 5 s prior to the end of the chase (figure 1). Thereafter, there was a significant decrease in speed (*χ*^2^ = 44.04, *p* < 0.001) but a significant increase in angular velocity (*χ*^2^ = 4.28, *p* = 0.039). Angular velocity differed with prey (*χ*^2^ = 27.58, *p* < 0.001) and, while chase speed was not related to hunt success (*χ*^2^ = 1.15, *p* = 0.284), angular velocity during the last 5 s was significantly greater when hunts were successful (*χ*^2^ = 13.44, *p* < 0.001). By comparison, the greatest mean VeDBA was 1.71 ± 1.10*g* and occurred 8 s prior to the end of the chase (figure 1). Thereafter, VeDBA decreased significantly (*χ*^2^ = 158.41, *p* < 0.001) and differed with prey species (*χ*^2^ = 40.96, *p* = 0.045).

**Figure 2.**
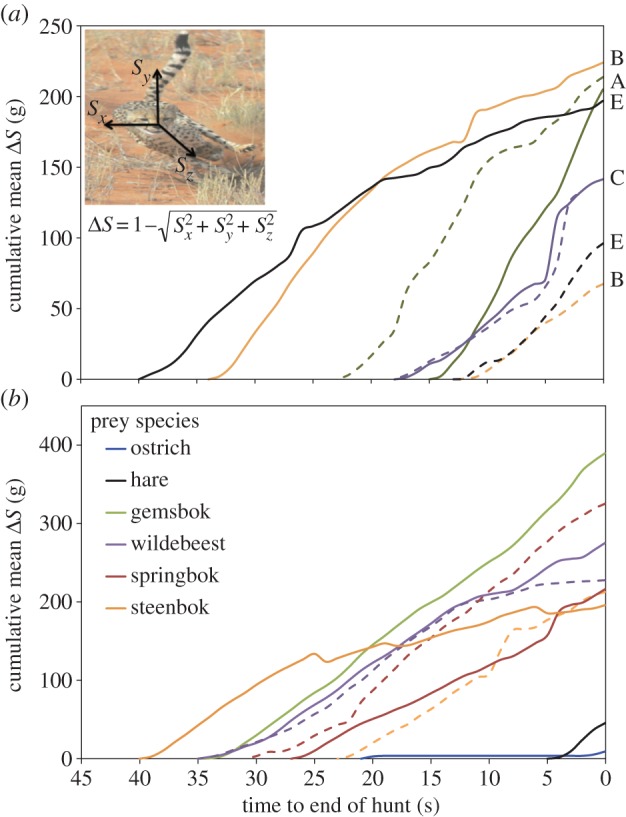
Mean cumulative VeSBA (*ΔS*, *g*) of successful (solid lines) and unsuccessful (dashed lines) cheetah hunts against time (*n* = 35, 30 Hz) for (*a*) steenbok hunts for cheetah individuals A–E and (*b*) different prey species chased. Mean *r*^2^ of linear regressions was 97.4 ± 2.75%, *S_x_*, *S_y_* and *S_z_* are orthogonal components of static acceleration.

The linear relationships of cumulative *ΔS* over time were significant (figure 2), indicating that forces exerted by the cheetahs resulted in approximately constant overall (lateral and forward) accelerations during chases. Over all chases, cheetahs were subject to an average *ΔS* of 0.27 ± 0.077*g* (max 0.45*g*). We observed significant three-way interactions between cheetah identity, hunt success and time as well as between prey species, hunt success and time on cumulative *ΔS* (*χ*^2^ = 1694.54, *p* < 0.001, figure 2*a* and *χ*^2^ = 358.29, *p* < 0.001, figure 2*b*). These results indicate that chase behaviour is both cheetah-specific and prey-specific (see electronic supplementary information, S2 and S3).

## 4. Discussion

Our results concur broadly with previous studies [2] in that, although the maximum speeds and acceleration values observed were impressive, and faster than racing greyhounds *Canis familiaris* (17.61 m s^−1^) [20], the values observed were slower than racehorses *Equus ferus* (19 m s^−1^) [21]. Thus, the widely held belief that cheetah hunts consist of simple high-speed chases seems an oversimplification. Rather, we suggest that cheetah chases comprise two primary phases: (i) an initial rapid acceleration resulting in high speed to quickly catch up with prey, followed by (ii) a prey-specific slowing period that enables the cheetah to match turns instigated by prey as the distance between them closes.

Mass-specific power of cheetahs during pursuit was recently estimated to reach 120 W kg^−1^ [2], astonishingly high compared with 30 W kg^−1^ of racehorses or 60 W kg^−1^ of greyhounds [22]. However, this formulation only considered forward acceleration and speed, ignoring lateral acceleration. In fact, Wilson *et al*. [16] demonstrated empirically that the lateral forces necessary for terrestrial animals to turn require considerable energy; walking humans executing a 180° turn require as much energy as 5.5 m straight-line travel. These lateral acceleration costs should be added to those derived from velocity and forward acceleration to obtain comprehensive power consumption figures. We thus suggest that a more likely reason why high turning angles and speeds do not co-occur ([2], figure 1) is at least partially power-based rather than being related to the capacity of limbs to withstand the forces generated [2] or environment conditions impeding the speeds seen in captive cheetahs running on a straight course [10]. A similar argument might suggest why in humans [23], as well as polo horses *Equus caballus* [15], maximum speed is limited by turn capability. In general, therefore, there is a trade-off between speed versus manoeuvrability in biological systems of which predator–prey hunting dynamics is one such pertinent example.

The varying amounts of force developed by the cheetahs chasing different prey, as shown in the cumulative *ΔS* plots (figure 2*b*), clearly illustrate species-specific chase strategies. If force generation is considered a major driver of power, this implies species-specific capture costs. Certainly, speed is only a part of the tactic; the ability to change direction rapidly to catch prey, such as small antelopes and ostriches that are adept at turning quickly, is also essential [9,13]. This critical capacity to turn, generally occurring during the final stages of the hunt, is at odds with high speeds, explaining the need for cheetahs to slow down. Indeed, this study shows that rapid turning during the final stages of the chase may be just as important and just as costly as accelerating rapidly at the beginning. Much of a cheetah's pursuit thus appears less of a high-speed rush, and more of a carefully played out life-or-death duel between predator and prey, in which opposing qualities of speed and manoeuvrability are pitted against each other.

## Notes

Permission and ethical clearance were granted by SANParks ethical and research committees to conduct the field research, Project Number 2006-05-10 MGMI. The study was performed in accordance with the commendations in the Guide for the Care and Use of Laboratory Animals of the National Institutes of Health. All immobilization and collaring of wild animals was conducted by a registered individual (GM), under the direction of a SANParks veterinarian.
